# A novel genetic system for recombinant protein secretion in the Antarctic *Pseudoalteromonas haloplanktis *TAC125

**DOI:** 10.1186/1475-2859-5-40

**Published:** 2006-12-14

**Authors:** Angela Maria Cusano, Ermenegilda Parrilli, Gennaro Marino, Maria Luisa Tutino

**Affiliations:** 1 Dipartimento di Chimica Organica e Biochimica, Università di Napoli Federico II – Complesso Universitario M.S. Angelo via Cinthia 4, 80126, Napoli Italia; 2 School of Biotechnological Sciences, Federico II University of Naples, Naples Italy

## Abstract

**Background:**

The final aim of recombinant protein production is both to have a high specific production rate and a high product quality. It was already shown that using cold-adapted bacteria as host vectors, some "intractable" proteins can be efficiently produced at temperature as low as 4°C.

**Results:**

A novel genetic system for the production and secretion of recombinant proteins in the Antarctic Gram-negative bacterium *Pseudoalteromonas haloplanktis *TAC125 was set up. This system aims at combining the low temperature recombinant product production with the advantages of extra-cellular protein targeting.

The psychrophilic α-amylase from *Pseudoalteromonas haloplanktis *TAB23 was used as secretion carrier. Three chimerical proteins were produced by fusing intra-cellular proteins to C-terminus of the psychrophilic α-amylase and their secretion was analysed. Data reported in this paper demonstrate that all tested chimeras were translocated with a secretion yield always higher than 80%.

**Conclusion:**

Data presented here demonstrate that the "cold" gene-expression system is efficient since the secretion yield of tested chimeras is always above 80%. These secretion performances place the α-amylase derived secretion system amongst the best heterologous secretion systems in Gram-negative bacteria reported so far. As for the quality of the secreted passenger proteins, data presented suggest that the system also allows the correct disulphide bond formation of chimera components, secreting a fully active passenger.

## Background

Either in the research community and biotechnology industry, *Escherichia coli *is the prokaryotic vector of choice for the high-level expression of proteins [1]. Unfortunately, this process sometimes results in the production of insoluble protein aggregates, incorrectly folded or non-functional proteins and proteins which may be degraded or contaminated with high levels of host-encoded proteins [2]. Since it has been reported that the lowering of the expression temperature can facilitate the correct folding of a "difficult" product [3,4], a new expression system [5] was recently developed which implemented the use of the Antarctic Gram-negative bacterium *Pseudoalteromonas haloplanktis *TAC125 (*P. haloplanktis *TAC125) [6] as host for protein production. By using such non-conventional system, some "intractable" proteins can be efficiently produced in soluble and active form at temperature as low as 4°C [7-9].

In general, bacteria secrete few proteins into the outside world. As such, protein secretion into the extra-cellular (outside) environment is the most desirable strategy; secreted proteins are not contaminated with other proteins and can be easily purified.

In this paper we report the setting up and use of a "cold" gene-expression system implemented for the secretion of recombinant proteins in *P. haloplanktis *TAC125. Such a system could effectively conjugate the positive effect of low temperature on the recombinant product solubility with the advantages linked to extra-cellular protein targeting.

This novel system makes use of the psychrophilic α-amylase from *P. haloplanktis *TAB23 [10] as secretion carrier. This exo-protein is synthesised as a preproenzyme, made of i) a Sec-dependent signal peptide; ii) a mature enzyme [11]; iii) a flexible spacer, and iv) a structurally independent C-terminal propeptide. The C-terminal propeptide is removed by the action of a host secreted protease which recognises and cleaves the -Ala-Ser-(↓)Ser-Thr- sequence contained in the flexible spacer. This event occurs when the precursor reaches the extra-cellular medium [12]. We demonstrated that the C-terminal propeptide is not mandatory for the *P. haloplanktis *TAB23 α-amylase recombinant secretion either in the source strain or in *P. haloplanktis *TAC125 [13]. Starting from the latter observation, it seemed interesting to study the secretion of chimerical proteins obtained by the replacement of α-amylase C-terminal propeptide with a passenger protein.

In this paper we describe the construction of a novel genetic system which allows the easy in frame cloning of any gene downstream of the mature psychrophilic α-amylase encoding region. Three chimerical proteins, obtained by fusing intra-cellular proteins to the psychrophilic exo-enzyme, were produced in *P. haloplanktis *TAC125 and their secretion was analysed. Results presented here demonstrate that the cold-adapted secretion system is efficient since all tested chimeras were translocated with a secretion yield always above 80%. Furthermore, activity data presented here indicate that the system also allows the correct disulphide bond formation of chimera components.

## Results

**Figure 1:**
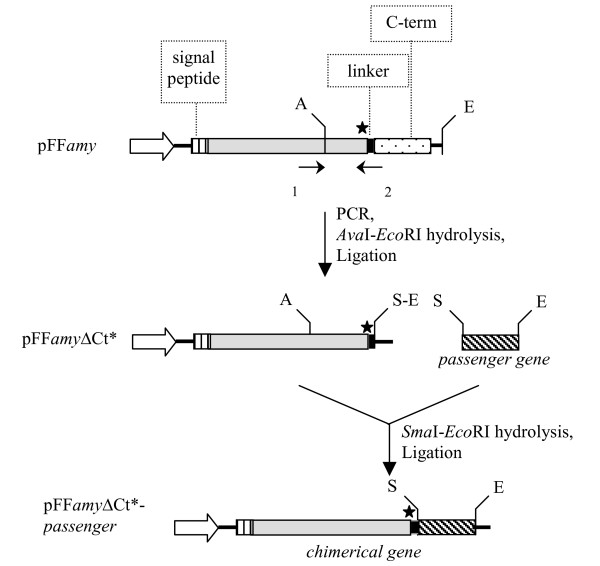
Construction of pFF*amyΔ*Ct* gene-expression vector and strategy for the construction of in-frame chimerical genes. White arrow, *P. haloplanktis *TAC125 *aspC *promoter; signal peptide, sequence encoding *P. haloplanktis *TAB23 α-amylase signal peptide; C-term, α-amylase C-terminal propeptide encoding sequence; linker, α-amylase linker encoding sequence; A, *AvaI*; E, *EcoRI*; S, *SmaI *restriction endonuclease sites; black arrows, PCR primers. The black star indicates the presence of a sequence encoding the amino acid motif -Ala-Ser-Ser-Thr-, recognised and cleaved by a *P. haloplanktis *TAC125 secreted protease.

Figure 1 describes the set up of the first genetic system for recombinant protein production and secretion in Antarctic bacteria. The pFF*amy *vector [13] was modified to remove the gene portion coding for α-amylase C-terminal propeptide; furthermore, two restrictions sites were introduced to allow in frame cloning just downstream of amylase linker encoding sequence. The flexible linker was conserved to allow the independent folding of the chimera's partners and their separation in the extra-cellular medium, due to the action of a *P. haloplanktis *TAC125 secreted protease, which recognises the linker sequence -Ala-Ser-Ser-Thr- and cleaves between the two Ser residues (unpublished results from this laboratory). The resulting generic vector was called pFF*amyΔ*Ct*.

Three protein passengers were used to test the versatility and efficiency of the psychrophilic recombinant secretion system set up: i) the hyper-thermophilic indole-3-glycerol-phosphate synthase (*Ss*IGPS) from *Sulfolobus solfataricus *(28 kDa) [14]; ii) the psychrophilic DsbA (*Ph*DsbA) from *Pseudoalteromonas haloplanktis *TAC125 (21 kDa) [15]; and iii) the mesophilic *Escherichia coli *β-lactamase (*Ec*BlaM) from the Tn3 transposon (31 kDa, Acc. No. EG10040). They are all monomeric and intracellular proteins: *Ss*IGPS is a cytoplasmic enzyme, while *Ph*DsbA and *Ec*BlaM are periplasmic proteins. A common strategy was applied for the construction of the chimerical genes (Figure 1). The passenger genes were PCR amplified to introduce *SmaI *and *EcoRI *restriction sites, and to remove the signal peptide encoding sequence in the case of *Ec*BlaM and *Ph*DsbA.

The resulting plasmids (pFF*amyΔ*Ct-*dsbA*, pFF*amyΔ*Ct-*trpC *and pFC*amyΔ*Ct-*blaM*) were mobilized into *P. haloplanktis *TAC125 by intergeneric conjugation [5]. Psychrophilic transconjugants were grown in liquid culture at 4°C, and samples were harvested at different phases during the growth.

**Figure 2:**
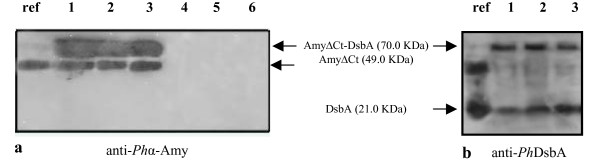
**Production and cellular localization of AmyΔCt-DsbA chimera in *P. haloplanktis *TAC125**. **a **Western Blotting analysis of extra-cellular media (lanes 1 to 3) and corresponding periplasmic extracts (lanes 4 to 6) of *P. haloplanktis *TAC125(pFF*amyΔ*Ct-*dsbA*) recombinant cells. Samples were collected during the growth at early, middle, and late exponential phases. The immunodetection was performed by using anti-α-amylase polyclonal antiserum. ref, AmyΔCt protein. **b **Western Blotting analysis of extra-cellular media (lanes 1 to 3) of *P. haloplanktis *TAC125(pFF*amyΔ*Ct-*dsbA*) recombinant cells performed using anti-*Ph*DsbA polyclonal antiserum. ref, Periplasmic extract of non-recombinant *P. haloplanktis *TAC125 cells, which contains endogenous DsbA.

Extra-cellular medium and corresponding periplasmic fractions of *P. haloplanktis *TAC125(pFF*amyΔ*Ct-*dsbA*) cells were analyzed using anti-*Ph*TAB23 α-amylase antiserum to evaluate production and cellular localization of the recombinant product. Western blotting analysis (Figure 2a) demonstrated that the AmyΔCt-DsbA chimera was produced in soluble form and localized in the extra-cellular medium (Figure 2a, lanes 1 to 3). As expected, the extra-cellular samples contained both AmyΔCt-DsbA and AmyΔCt proteins, as a result of proteolytic cleavage of chimera linker. To confirm that the extra-cellular targeting of the recombinant products was due to a specific secretion mechanism, the integrity of the host outer membrane was evaluated by monitoring the presence of endogenous periplasmic alkaline phosphatase. As shown in Table 1, alkaline phosphatase activity was almost totally retained into the periplasmic fraction, thus ruling out the occurrence of a unspecific cell leakage.

**Table 1:** Secretion yield of chimerical proteins in recombinant *P. haloplanktis *TAC125 cells

vector	α-amylase (UI/ml)		α-amylase	alkaline phosphatase	**secretion yield**^**a**^
	p	em	em(%)	em(%)	
pFF*amyΔ*Ct-*dsbA*	0.12 ± 0.01	3.58 ± 0.03	97	4	**93**
pFF*amyΔ*Ct-*trpC*	0.07 ± 0.02	3.65 ± 0.03	98	5	**93**
pFF*amyΔ*Ct-*blaM*	1.23 ± 0.03	6.21 ± 0.03	83	1	**82**

Data are average results of three independent experiments. The volume of periplasmic fraction was made the same as corresponding extra-cellular medium to allow a comparison. UI, international units; p, periplasmic extract; em, extra-cellular medium fraction; em(%), percentage of the activity in extra-cellular medium fraction of the total activity (periplasmic plus extra-cellular medium fractions).

^a ^The difference between the em (%) of amylase activity and em (%) of alkaline phosphatase activity.

The *P. haloplanktis *TAC125(pFF*amyΔ*Ct-*dsbA*) extra-cellular samples were further immunodetected by anti-*Ph*DsbA antisierum (Figure 2b). As control, a periplasmic extract of non-recombinant *P. haloplanktis *TAC125 cells was analysed (Figure 2b, lane ref), which contains the endogenous DsbA. The polyclonal antiserum recognised two proteins, one corresponding to the AmyΔCt-DsbA chimera and the other one accounting for the free passenger DsbA. Taken together, results presented in Figure 2a and 2b demonstrated that the culture supernatants contain the AmyΔCt-DsbA chimera and its free components (i.e. the carrier α-amylase and the passenger *Ph*DsbA) deriving from a proteolytic cleavage in chimera linker.

The recorded psychrophilic α-amylase activity (accounting for either the chimerical enzyme or the free one) was used to calculate the secretion yield, which resulted to be above 90% (Table 1). The *Ph*DsbA catalytic activity was not detectable since the highest DsbA production (1.8 mg/l) resulted to be below of the DsbA catalytic assay sensitivity [16].

**Figure 3:**
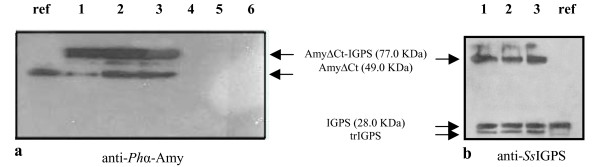
**Production and cellular localization of AmyΔCt-IGPS chimera in *P. haloplanktis *TAC125**. **a **Western Blotting analysis of extra-cellular media (lanes 1 to 3) and corresponding periplasmic extracts (lanes 4 to 6) of *P. haloplanktis *TAC125(pFF*amyΔ*Ct-*trpC*) recombinant cells. Samples were collected during the growth at early, middle, and late exponential phases. The immunodetection was performed by using anti-α-amylase polyclonal antiserum. ref, AmyΔCt protein. **b **Western Blotting analysis of extra-cellular medium (lanes 1 to 3) of *P. haloplanktis *TAC125(pFF*amyΔ*Ct-*trpC*) performed using anti-*Ss*IGPS polyclonal antiserum. trIGPS, truncated form of *Ss*IGPS. ref, *Ss*IGPS protein.

A similar approach was applied to analyze production and cellular localization of the AmyΔCt-IGPS chimera in *P. haloplanktis *TAC125(pFF*amyΔ*Ct-*trpC*) cells. As shown in Figure 3 panel a, the chimera was largely secreted in the extra-cellular samples (lanes 1 to 3, and Table 1) and the AmyΔCt-IGPS linker was partially cleaved, releasing AmyΔCt protein. Recombinant extra-cellular samples were further immunodetected using anti-*Ss*IGPS antiserum and results are shown in Figure 3, panel b. The samples turned out to contain AmyΔCt-IGPS, the free *Ss*IGPS (as compared to the *Ss*IGPS loaded in lane ref), and a stable truncated *Ss*IGSP form (trIGPS). The latter product likely is due to an unexpected sensitivity of the passenger protein to host encoded extra-cellular proteases. However, no thermophilic *Ss*IGPS activity was detected in the culture medium.

**Figure 4:**
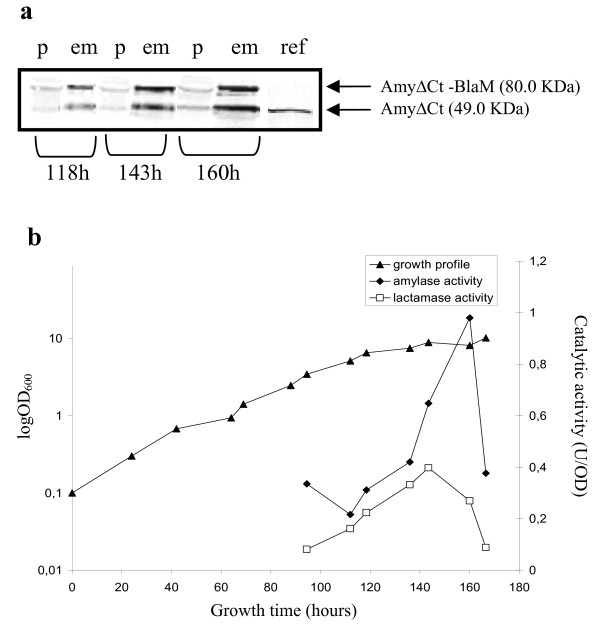
**Production, cellular localization and enzymatic activities of AmyΔCt-BlaM chimera in *P. haloplanktis *TAC125 recombinant cells**. **a **Western Blotting analysis of extra-cellular media (lanes 2, 4, 6) and corresponding periplasmic extracts (lanes 1, 3, 5) of *P. haloplanktis *TAC125(pFF*amyΔ*Ct-*blaM*) recombinant cells. Samples were collected at the indicated times. The immunodetection was performed by using anti-α-amylase polyclonal antiserum. ref, AmyΔCt protein. **b **(▲) *P. haloplanktis *TAC125(pFF*amyΔ*Ct-*blaM*) liquid growth profile, (◆) α-amylase enzymatic activity, and (□) β-lactamase enzymatic activity recovered in the extra-cellular medium.

*P. haloplanktis *TAC125(pFC*amyΔ*Ct-*blaM*) cells produced and secreted the AmyΔCt-BlaM chimera as demonstrated by immunoblotting using anti-α-amylase antiserum (Figure 4a). The chimera's linker was cleaved as occurred for the other tested chimeras releasing AmyΔCt and the free passenger. β-Lactamase and α-amylase activities were assayed on culture medium samples collected during growth phase and the resulting activity profiles are shown in Figure 4b. These data were used to calculate the molar ratio between the α-amylase and β-lactamase accumulated in the extra-cellular samples. The ratio remains roughly equal to 1:1 till 140 hours (data not shown), suggesting that the passenger was fully active either in chimerical or in free form. After 140 hours of growth, a reduction in recorded β-lactamase activity was observed. The higher secretion yield was achieved at the beginning of stationary phase as shown by the secretion kinetics (Figure 4b).

## Discussion and Conclusion

The aim of recombinant protein production is to achieve both a high specific production rate and a high product quality. One strategy to avoid quality problems and improve protein production is to target the protein to outer compartments of the host cell [17]. This strategy allows to avoid inclusion body formation and to achieve a primary purification reducing the costs of downstream processes.

In this paper we report the use of a cold-adapted α-amylase as secretion carrier for the extra-cellular protein targeting by the Antarctic marine bacterium *P. haloplanktis *TAC125. Efficiency and versatility of this novel genetic system was probed with three passenger proteins, that display different molecular properties. As previously reported for the psychrophilic α-amylase [13], secretion of AmyΔCt-derived chimerical proteins requires the crossing of two membranes, and the transit into the periplasmic space, where protein folding and disulphide bond formation can occur.

The Sec-dependent translocation of all the tested chimeras turned out to be always complete, since no fusion product was ever detected into the recombinant cytoplasmic extracts (data not shown). The following translocation step (i.e. from periplasmic space to the extra-cellular medium) occurs by a still uncharacterized secretion machinery [6,18] and it resulted to be only slightly less efficient, since the secretion yield of tested chimeras is always above 80% (Table 1). These secretion performances place the α-amylase derived secretion system amongst the best heterologous secretion systems in Gram-negative bacteria reported so far [19-21]. As for the quality of the secreted passenger proteins, activity data presented demonstrate that, at least in the case of the mesophilic β-lactamase, the system allows its correct folding, secreting a fully active passenger.

However, results presented in this paper address to a potential limit of the newly set up recombinant secretion system: host extra-cellular medium contains proteolytic activities which can inactivate some heterologous products. For instance, *Ss*IGPS displays two exposed loops, located at the N-terminal region, accessible to the action of a host protease [22]. If a single cleavage occurs in this region the activity of truncated enzyme results to be affected [23]. This evidence can justify the absence of *Ss*IGPS enzymatic activity in the extra-cellular samples of *P. haloplanktis *TAC125(pFF*amyΔ*Ct-*trpC*) cells. The exo-protease action on passenger protein can also justify the shift between the maximum of α-amylase activity with respect to the maximum of β-lactamase activity after 140 hours of growth (Figure 4b). It is reasonable that secreted proteases accumulate in stationary phase and could interfere with β-lactamase stability.

To overcome this problem, it would be useful to develop a novel *P. haloplanktis *TAC125 mutant which secretes a reduced number of exo-proteases. This approach has recently became feasible thanks to the publication of *P. haloplanktis *TAC125 genome [6]. Indeed, beside giving some insights into the specific strategies adopted by *P. haloplanktis *TAC125 to grow at low temperature, the genome knowledge is instrumental to set up a suitable scheme for genome engineering.

The genetic system presented in this paper further increases the number of reliable genetic tools already set up in *P. haloplanktis *TAC125 [5,8,9,24], making concrete the use of this Antarctic marine bacterium as non-conventional host for the production of "difficult" proteins, which are not successfully expressed in any other expression systems.

## Methods

### Strains and plasmid

*P. haloplanktis *TAC125 was isolated from Antarctic sea water [6]. *Escherichia coli *DH5α [25] was used as host for the gene cloning.

The chimeric *amyΔ*Ct-*dsb *A gene was made by fusing the *P. haloplanktis *TAC125 *dsbA *gene [15] to the 3' end of the of the *amyΔ*Ct gene. As shown in Figure 1, the 3' region of the *amy *gene was amplified to remove the DNA sequence coding for C-terminal propeptide, and to introduce *SmaI *and *EcoRI *restriction sites (primers 1–2 5'-CGCCAGGGTTTTCCCAGTCACGAAC-3' and 5'-GTGAATTCCCAGTCGACCCGGGTGCTTGAGGCAGAACTGG-3'). The PCR product was subjected to a double *Ava*I and *EcoR*I digestion and inserted into pFF*amy *[13] corresponding sites, generating pFF*amyΔ*Ct* (Figure 1). The *dsbA *gene was amplified by PCR to remove its signal peptide encoding sequence and to introduce *Sma*I and *Eco*RI restriction sites using primers 3–4 (5'AACCCGGGCAAACTTTGAAGTAGG3' 5'TTTGAATTC CAAAAATTTATAG 3'). The PCR product was subjected to a double *Sma*I and *Eco*RI digestion and inserted into pFF*amyΔ*Ct* corresponding sites, generating pFF *amyΔ*Ct-*dsbA*.

The chimeric *amyΔ*Ct-*trpC *gene was constructed by fusing the *Sulfolobus solfataricus trpC *gene [14] to the 3' end of the of the *amyΔ*Ct* gene. The *trpC *gene was amplified by PCR to introduce*Sma*I and *Eco*RI restriction sites (primers 5–6, 5'GGAATGTCGACCTGCAGATGCCACGTTATCTTAAAGGATGG3' 5'CCCGAGCTCAGGTACCTAGTATGAATTCTTTAATCTTTTC3'), and resulting PCR product was subjected to a double *Sma*I and *Eco*RI digestion and inserted into pFF*amyΔ*Ct* corresponding sites, generating pFF*amyΔ*Ct-*trpC*.

The pFC*amyΔ*Ct-*blaM *plasmid was constructed as previously reported [18]; it contains the *blaM *gene (acc no. EG10040) which was amplified by PCR to remove its signal peptide coding sequence. It is also characterized by the presence of chloramphenicol resistance marker.

All PCR amplifications were performed as described [26]. The amplified fragments were cloned and their nucleotide sequences checked to rule out the occurrence of mutations during synthesis.

### Growth condition and analytical procedure

*P. haloplanktis *TAC125 was grown in aerobic conditions at 4°C in TYP broth (16 gr/l yeast extract, 16 gr/l bacto tryptone, 10 gr/l sea salts) at pH 7.5, supplemented with ampicillin 200 μg/ml or chloramphenicol 25 μg/ml, if transformed. Antarctic bacteria transformation was achieved by intergeneric conjugation as previously reported [5].

*E. coli *cells were routinely grown in Terrific broth [26]containing 100 μg/ml of ampicillin or clorampheincol 50 μg/ml, if transformed.

The extraction of periplasmic proteins was performed by osmotic shock as previously described [15]. Protein samples for Sodium Dodecyl Sulfate-Polyachrylamide Gel Electrophoresis were prepared and separated on SDS-containing polyacrylamide (12%) gels using standard methods [26]. For immunoblotting, the gels were transferred to a polyvinylidene difluoride membrane (Immobilon PSQ, Millipore). For immunodetection of proteins, *P. haloplanktis *TAB23 anti-α-amylase [12], *P. haloplanktis *TAC125 anti-DsbA [15], and *S. solfataricus *anti-IGPS antisera were diluted in blocking buffer (phosphate buffer saline; 5% skimmed milk). Peroxidase conjugate anti-rabbit IgG (Sigma-Aldrich, USA) was used as secondary antibody. Proteins were detected by chemioluminescence (Pierce, USA).

α-Amylase activity was assayed by using the Boehringer-Roche kit AMYL in the conditions previously reported [12]. Alkaline phosphatase activity was assayed according to [27]. β-Lactamase activity was assayed according to [28]. *S. solfataricus *IGPS enzymatic activity was assayed according to [14]. *P. haloplanktis *TAC125 DsbA activity was tested as previously reported [15].
